# Four-Days of Passive Heat Acclimation Increases Exercise Capacity in Healthy Older Adults Living in the UK

**DOI:** 10.3390/healthcare14081005

**Published:** 2026-04-11

**Authors:** Laura J. Wilson, Emma V. Ward, Luke W. Oates

**Affiliations:** 1 London Sport Institute, Middlesex University, London NW4 4BT, UK; l.oates@mdx.ac.uk; 2 Department of Psychology, Middlesex University, London NW4 4BT, UK; e.ward@mdx.ac.uk

**Keywords:** hot water immersion, heat related illness, thermal strain, ageing

## Abstract

**Highlights:**

**What are the main findings?**

- Four days of hot water immersion increased exercise capacity (*p* = 0.028, d = 0.89).
- Thermal sensation and comfort ratings remained unchanged post-intervention.

**What are the implications of the main findings?**

- Heat acclimation strategies may offer protection against heat related illness.
- Passive strategies may be a feasible intervention for vulnerable individuals.

**Abstract:**

**Background:** Older adults are particularly vulnerable to heat related illness due to impaired thermoregulatory responses. Heat acclimation (HA) strategies can mitigate the negative impacts of high environmental temperatures on physiological and perceptual responses. Whilst active HA strategies may prove problematic for older adults, passive approaches such as hot water immersion (HWI) may be more feasible. **Methods:** This study investigated the effects of four consecutive days of HWI on physiological and perceptual markers in individuals aged over 65 years during moderate exercise. Nine healthy, recreationally active participants (76 ± 5 years) completed two 30 min cycling bouts at 75–80% age predicted HR_max_ pre- and post-four days of HWI at 40 °C. Measures of average HR, gastrointestinal temperature, skin temperature, thermal sensation, thermal comfort, rate of perceived exertion, power output, and distance covered were recorded during both exercise bouts. **Results:** Results showed a significant increase in exercise capacity as measured by power output (*p* < 0.05, 7.45 W) post-intervention, despite no change in ratings of perceived exertion, and reductions in average heart rate (112 ± 3 vs. 109 ± 4 bpm). There were no alterations in gastrointestinal or skin temperature, and ratings of thermal comfort and sensation remained unchanged post-intervention. **Conclusions:** These preliminary findings provide important new evidence that four days of passive HWI may be a practical and effective method of inducing physiological adaptations in older individuals, which may be of use in interventions to mitigate the negative impact of high environmental temperatures in this population.

## 1. Introduction

Climate change is driving significant increases in mean global temperatures [1]. As temperatures continue to rise, severe adverse climactic phenomena such as heat waves are becoming more common. In the UK, a heatwave is defined as at least three consecutive days in which the daily maximum temperature reaches or exceeds a specific temperature threshold for recognised counties of the United Kingdom (ranging between 25 and 28 °C) [2]. In addition to growing frequency, the duration and intensity of heat waves is also increasing [1]. At the same time, the proportion of individuals vulnerable to heat-related illness is also rising. In particular, the world is experiencing a shift in population demographics in relation to age. In 2021, the number of individuals in the UK aged 65 and over exceeded 11 million, representing 18.6% of population [3]. This figure is projected to increase such that by 2050 individuals over the age of 65 will account for approximately 25% of the UK population [4]. Whilst life expectancy is increasing, research suggests that ‘healthy life years’ are not rising at the same rate [5], indicating that the number of people vulnerable to heat-related illnesses as a result of advancing age and/or pre-existing comorbidities could continue to rise drastically. Older individuals are at an elevated risk of heat-related illness for a number of reasons, including a diminished ability to regulate internal physiological systems that dissipate heat (impaired sweat response), reduced skin blood flow, limited mobility, blunted perceptual responses during exercise, or a diminished thirst response [6]. The intersectionality of increased age, co-existing conditions, and medication that may inhibit heat dissipation, increases the risk of adverse health effects arising from exposure to extreme temperatures [7]. Indeed, there is evidence that heat waves are associated with increased mortality rates in older individuals [8], with greater increases observed during more intense heat waves [9].

Given the well-documented links between increased temperatures and negative health outcomes [7,8,9], it is unsurprising that there is a considerable body of literature examining the impact of heat acclimation (HA) strategies on physical and cognitive markers of performance. However, much of this research has been conducted in athletic populations, or those exposed to extreme temperatures through their jobs (e.g., soldiers and firefighters). There is strikingly little research on the effects of HA on older individuals [10]; however, the available evidence suggests that HA can elicit reductions in core temperature [11,12,13] or skin temperature [11,14] during exercise, increase sweat rates [12,14,15,16], and reduce body heat storage [12]. However, considerable gaps still exist, and further research is needed to identify safe and practical HA strategies for this population.

HA strategies and protocols are extremely adaptable, with variations in duration (both in terms of individual sessions and number of days of heat exposure), session arrangement (consecutive versus non-consecutive exposures), temperature, humidity, and activity (active versus passive protocols) reported in the literature [17]. By repeatedly inducing hyperthermia (core temperature > 38.5 °C) under controlled conditions, it is possible to bring about physiological adaptations that can reduce thermal strain. These beneficial adaptations include reduced resting and exercising core temperature, reduced heart rate, and increased sweat rate, all of which can enhance functional capacity [10]. Whilst medium- and long-term heat acclimation strategies (e.g., 7–14 days and over 14 days, respectively) are likely to offer the most complete adaptive response, short-term heat acclimation (STHA) strategies of between four and seven days can elicit ~80% of the same adaptation and are more practical and logistically feasible to implement [18].

Active, exercise-based HA protocols are commonly utilised, and are often implemented in conjunction with an environmental chamber to adjust ambient temperature. However, this approach is likely not appropriate for individuals who may already be considered more susceptible to heat-related illnesses, those with contra-indications to intense exercise, such as older individuals [10], or practical for use in the real-world due to availability of heat chambers. However, passive HA strategies, such as hot water immersion or the use of saunas or heated garments, can induce physiological adaptations similar to those elicited by exercise [10], providing a more practical and appropriate intervention for older individuals. The time course of HA is remarkably rapid, with most adaptations occurring during the first week of heat exposure [19]. However, it has previously been demonstrated that three days of passive HA is insufficient to elicit physiological adaptations in young or elderly females [20]. Thus, determining the shortest effective duration will allow for the effective and efficient implementation of HA protocols.

The combination of an ageing population and more frequent heat waves due to climate change means that there is a strong need to seek out strategies to help vulnerable individuals minimise physiological stress associated with activities of daily living during times of increased environmental temperatures [21]. Therefore, the aim of this study was to investigate the impact of a four-day passive HA strategy on physiological and perceptual markers in individuals aged over 65 years during moderate intensity cycling exercise designed to reflect the demands of activities of daily living. Outcome measures were taken while participants engaged in moderate exercise in thermoneutral conditions before and after 4 days of passive heating via hot water immersion.

## 2. Materials and Methods

### 2.1. Participants

Healthy and recreationally active participants aged over 65 years of age were recruited from the University of the 3rd Age (U3A), through targeted emails and word of mouth. The recruitment period for the study spanned from 31 May 2023 until 31 January 2024. All participants completed a health screen questionnaire based on the validated PAR-Q [22], with additional health history questions and study specific exclusions. A copy of the questionnaire is included as a Supplementary File. Participants were required to be regularly active (including walking). If participants had ever been advised by their GP or other medical professional not to partake in strenuous activity, they were excluded from the study. Individuals with contra-indications to the study procedures (swallowing/digestive issues, gastrointestinal disorders, sensitivity to chlorine, circulatory problems, intolerance to heat, or contra-indications to the core temperature pills) were ineligible to participate. Sample sizes in prior studies in this area range from 6 to 10 older participants [10], so a minimum sample size of 8 participants was determined. Participants were recruited using convenience sampling. Ten participants completed the study in full, but one was excluded prior to analysis due to their exceptionally high training status (average power output >300% of the group mean value; 163 v 51.33 W at baseline respectively), which was not representative of the population of interest. The final sample comprised nine individuals (4 female) with a mean age of 76 years (SD = 5; range = 69–84 years). Physical characteristics for the sample are given in Table 1. The research conformed to the standards set by the Declaration of Helsinki. Ethical approval was granted by the Middlesex University Research Ethics Committee, and all participants provided written informed consent prior to taking part in the study.

### 2.2. Design and Procedure

**Figure 1:**
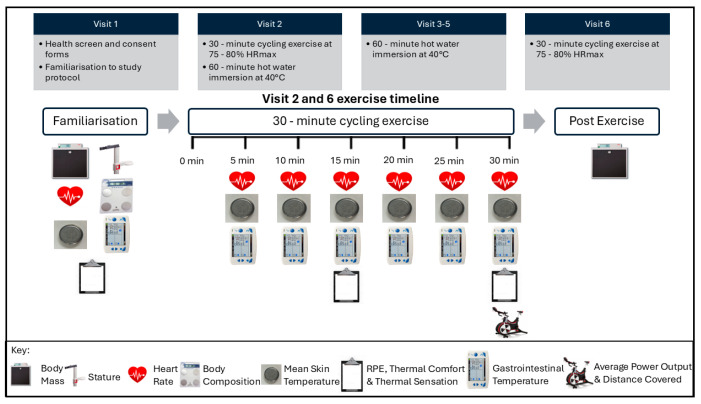
Study overview and timeline, including exercise trial measurements on visits 2 and 6.

Participants attended a total of six sessions, and the testing procedures are outlined in Figure 1. Following the familiarisation, visits 2 to 6 were conducted on consecutive days, with pre-intervention measures recorded at visit 2, and post-intervention measures recorded at visit 6. Laboratory conditions were standardised for each visit, with temperature maintained at 22.1 ± 1.4 °C. This temperature was chosen on the basis that 20–24 °C is considered comfortable for most individuals, especially vulnerable groups [22]. Measures of thermal sensation (TS), thermal comfort (TC), mean skin temperature (T_skin_), gastrointestinal temperature (T_gast_), rating of perceived exertion (RPE), average heart rate (HR), average power output (PO), and distance covered in kilometres on a cycle ergometer, were collected during exercise in thermoneutral conditions before and after 4 days of passive heating applied via hot water immersion (HWI). The fixed intensity exercise bouts represented active heat stress to replicate activities of daily living and were completed in thermoneutral conditions, in order to test the feasibility and efficacy of the HA strategy.

### 2.3. Anthropometric Measurements

On visit 2 participants’ height and mass were recorded to the nearest 0.1 cm and kg, respectively, using a stadiometer and scale (Seca, 213 and 875, Medisave UK, Dorset, UK). Using this information, body mass index (BMI) was calculated, and then body fat percentage (BF%) was estimated to the nearest 0.1% using Tanita Bioelectrical Impedance Scales (Tanita, SC-240MA, Amsterdam, The Netherlands). Each participant’s sex, age in years, and height in cm was entered into the scales. Participants then stepped onto the scales ensuring their weight was distributed evenly between both feet and ensuring contact with all four electrode surfaces (ball and heel of each foot).

### 2.4. Gastrointestinal Temperature

At the familiarisation session (visit 1), participants were provided with a programmed ingestible telemetric core temperature pill (e-Celsius performance, BodyCAP, Hérouville Saint-Clair, France), and instructed to swallow the pill with water on the morning of their pre-intervention testing session (visit 2) a minimum of 2 h prior to arrival as per manufacturer instructions. This ensured sufficient time for the pill to enter the digestive tract for accurate gastrointestinal (T_gast_) measurements. Participants were given a second pill at the end of their 5th visit to ingest the following morning before the final exercise session (visit 6). The e-Celsius pills have previously been shown to demonstrate excellent validity, and test–retest reliability for T_gast_ between 36 °C and 44 °C [23]; an external wireless recorder was used to receive the signal from the temperature capsule via a specific radio frequency. The data recorder was held in close proximity to participants (2–3 cm away from the skin of the lower back or abdomen) to ensure that the pills were recording correctly before commencement of each exercise session. All data was downloaded onto the wireless recorder at the end of the 2nd and 6th visit for analysis. Measures of T_gast_ were recorded immediately prior to the exercise bout and at 5 min intervals until exercise cessation.

### 2.5. Mean Skin Temperature

Mean skin temperature (T_skin_) was assessed utilising a wireless Thermochron iButton^®^ (DS1922) system (Maxim Integrated, San Jose, CA, USA). Thermochrons were attached to the pectoralis major, biceps brachii, rectus femoris and gastrocnemius on the right side of the body in line with previous investigations [24], were set to record every minute during exercise, and secured to the skin with hypoallergenic adhesive bandage. Following cessation of exercise, the iButtons (e-Celsius performance, BodyCAP, Hérouville Saint-Clair, France) were removed and data was downloaded for analysis. Measures of T_skin_ were recorded immediately prior to the exercise bout and at 5 min intervals until exercise cessation. Mean skin temperature was determined as (0.3 × (B + C)) + 0.2 × ((T + Cf)), where B is bicep temperature, C is chest temperature, T is thigh temperature, and Cf is calf temperature [25].

### 2.6. Exercise Protocol

Participants were required to complete a 30 min exercise bout on a WattBike Pro cycle ergometer (WattBike Ltd., Nottingham, UK), whilst maintaining 75–80% of age predicted heart rate maximum (HR_max_). Appropriate ranges were calculated using the ACSM formula for age predicted HR_max_ [22]. Whilst it is acknowledged that prediction equations may overestimate true maximum HR in certain populations, this was chosen to avoid subjecting participants to a maximal exercise protocol in order to determine true HR_max_. The protocol allowed participants to modulate intensity to match their individualised HR targets, and is comparable to similar studies undertaken previously in this population. Participants were permitted to drink ad libitum during the exercise. The same exercise protocol was undertaken by each participant on 2 separate occasions, pre- and post-intervention (visit 2 and visit 6; see Figure 1). Following the exercise bout, participants were asked to towel themselves dry if necessary, and a second measure of body mass was taken to allow for a fluid loss calculation to be completed. Average power output and total distance covered was recorded at the end of exercise.

### 2.7. Thermal Sensation and Comfort

**Figure 2:**
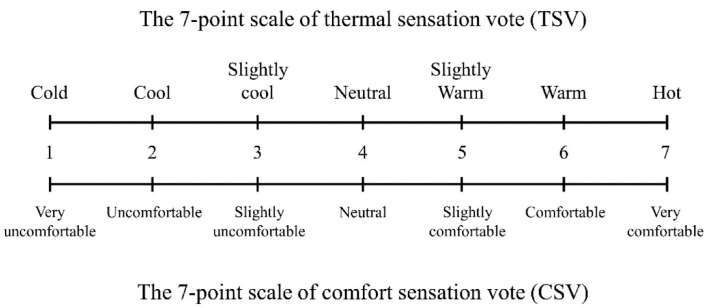
Thermal sensation and thermal comfort scale.

Measures of thermal sensation (TS) and thermal comfort (TC) were recorded immediately before, at the midpoint (15 min), and at the end (30 min) of the exercise bout. Both variables were measured using a 7-point Likert scale with text descriptors for each point [26] (Figure 2).

### 2.8. Heart Rate

Participants were fitted with a chest worn heart rate (HR) monitor (Polar H10, Polar, Kempele, Finland) which was synced with a tablet device running Polar Team software 4.0.4. This allowed for continuous simultaneous display of live HR data and percentage of age predicted HR_max_ to enable participants to maintain the required exercise intensity for the duration of the exercise protocol (75–80% age predicted HR_max_). Average HR during the exercise bout was recorded.

### 2.9. RPE

Participants’ rating of perceived exertion (RPE) was recorded at 15 min and 30 min during the exercise protocol, using a 6–20 scale [27].

### 2.10. Immersion Protocol

Participants completed the immersion protocol on visits 2–5. They were submerged to mid-axillary level in a hot water immersion tank (Ibiza Airjet, LAY-Z-SPA, Bestway, London, UK) heated to 40 °C, for 60 min. The temperature was automatically monitored and maintained by the integrated heating pump unit throughout each immersion. The immersion duration was based on evidence that it takes ~15 min for core temperature to reach 38.5 °C, and that a minimum of 30 min exposure above 38.5 °C is required to elicit adaptive responses [10].

### 2.11. Data Analysis

The de-identified raw data is openly available at https://osf.io/5acjt/ (access on 30 January 2025). Analyses were performed using SPSS 27.0. Following confirmation of normal distribution using Shapiro–Wilk tests, the effect of Intervention (pre-post) was examined for each variable using paired *t* tests (body mass, power output, average heart rate, and distance), or repeated measures ANOVAs (thermal sensation, thermal comfort, rate of perceived exertion, gastrointestinal temperature, and skin temperature). An alpha level of 0.05 was used for all statistical tests, and all t tests were two-tailed. Where the assumption of sphericity was violated, Greenhouse-Geisser adjusted degrees of freedom and probability levels are reported. Partial eta squared (ηp^2^) effect sizes are provided for ANOVA effects and Cohen’s d for t tests [28]. The ηp^2^ value was interpreted as ηp^2^ < 0.01 for trivial, 0.01 ≤ ηp^2^ < 0.06 for small, 0.06 ≤ ηp^2^ < 0.14 for medium, and 0.14 ≤ ηp^2^ for large effects. When a multiple comparison test was performed, Cohen’s d was calculated, and interpreted as d < 0.20 for trivial, 0.20 ≤ d < 0.49 for small, 0.50 ≤ d < 0.8 for medium, and 0.8 ≤ d for large effects.

## 3. Results

Table 2 summarises all measures recorded pre-intervention and post-intervention.

### 3.1. Heat Acclimation

**Figure 3:**
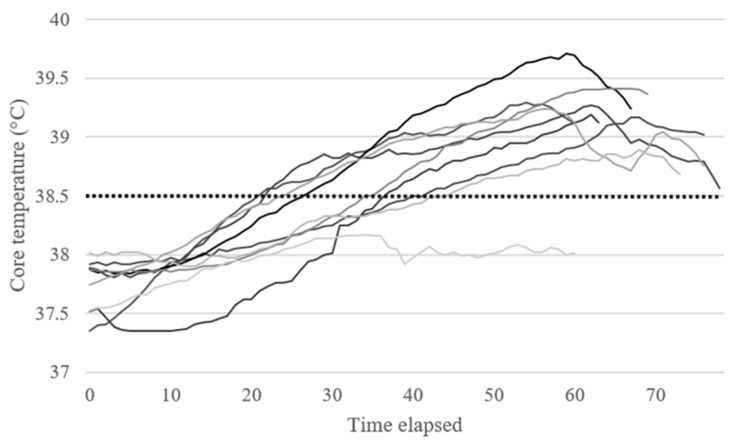
Series lines represent individual core temperature data for the first HWI session. Elapsed time represents time from entering the hot water immersion tank until the core temperature pill was deactivated post-immersion. All immersions were 60 min in total.

All participants tolerated the HA protocol and completed each immersion session as prescribed. Data from the ingestible temperature pills revealed that the HA protocol was effective for eliciting a core temperature of ≥38.5 °C for 8 out of 9 participants during the first immersion. Anecdotal evidence from the 9th participant revealed that they regularly spent time in hot tubs, which indicates that they were already well adapted to passive heat exposure. Previous research indicates that a minimum of 30 min at a core temperature of ≥38.5 °C is required to elicit adaptive responses [10]. In the current study, participants experienced a core temperature at or above 38.5 °C for 35.33 ± 16.42 min as a result of the first immersion session, and this increased to 39.75 ± 10.44 min when the outlier was removed (Figure 3).

Analysis of Exercise Performance: Physiological and Perceptual Markers

### 3.2. Body Mass

Body mass (kg) was measured pre-intervention and again following the intervention, both before and after exercise, in order to assess fluid loss, with any ad libitum fluid intake factored into this equation. A 2 × 2 repeated measures ANOVA revealed a marginal main effect of exercise (pre vs. post exercise), F(1, 8) = 5.26, *p* = 0.051, ηp^2^ = 0.396, but no main effect of intervention (pre vs. post intervention), F(1, 8) = 2.20, *p* = 0.176, ηp^2^ = 0.216, and no interaction between the two, F(1, 8) = 0.05, *p* = 0.824, ηp^2^ = 0.007.

### 3.3. Heart Rate, Power Output, and Distance

**Figure 4:**
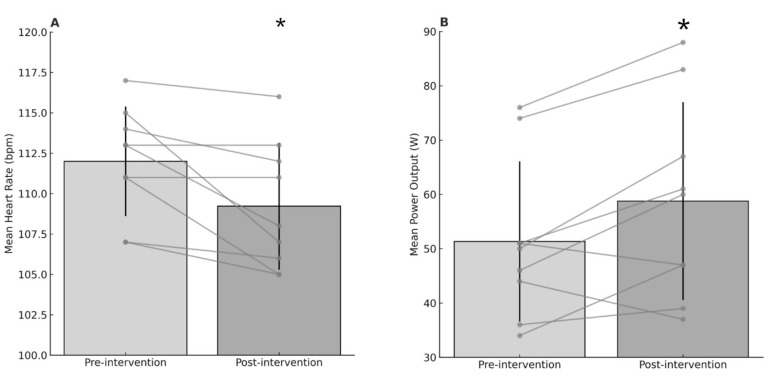
(**A**): Mean heart rate (bpm) pre-intervention and post-intervention. (**B**): Mean power output (W) pre-intervention and post-intervention. Error bars indicate standard deviation of the mean, and circles indicate individual data points. * Significantly different from pre-intervention.

Pre-post intervention there was a significant reduction in heart rate (Figure 4A), t(8) = 2.91, *p* = 0.020, d = 0.97, a significant increase in power output (Figure 4B), t(8) = 2.68, *p* = 0.028, d = 0.89, but the increase in distance covered was not significant, t(8) = 1.22, *p* = 0.257, d = 0.41. Sensitivity analyses revealed that removal of the excluded participant did not alter the pattern of responses nor direction of change for the exercise variables.

### 3.4. Thermal Sensation and Thermal Comfort

A 2 × 3 repeated measures ANOVA showed a significant main effect of time on thermal sensation, F(2, 16) = 54.25, *p* < 0.001, ηp^2^ = 0.871, indicating an increase in thermal sensation over the course of the exercise. However, there was no main effect of intervention, F(1, 8) = 2.29, *p* = 0.169, ηp^2^ = 0.222, and no interaction, F(1.21, 9.67) = 0.61, *p* = 0.494, ηp^2^ = 0.071. On thermal comfort there was no main effect of Time, F(2, 16) = 1.46, *p* = 0.262, ηp^2^ = 0.154, no main effect of intervention, F(1, 8) = 3.56, *p* = 0.096, ηp^2^ = 0.308, and no interaction, F(2, 16) = 0.34, *p* = 0.720, ηp^2^ = 0.040.

### 3.5. Ratings of Perceived Exertion

A 2 × 2 repeated measures ANOVA showed no main effect of time, F(1, 8) = 0.76, *p* = 0.409, ηp^2^ = 0.087, no main effect of intervention, F(1, 8) = 1.00, *p* = 0.347, ηp^2^ = 0.111, and no interaction, F(1, 8) = 0.31, *p* = 0.594, ηp^2^ = 0.037.

### 3.6. Analysis of Changes in Gastrointestinal and Skin Temperatures

**Figure 5:**
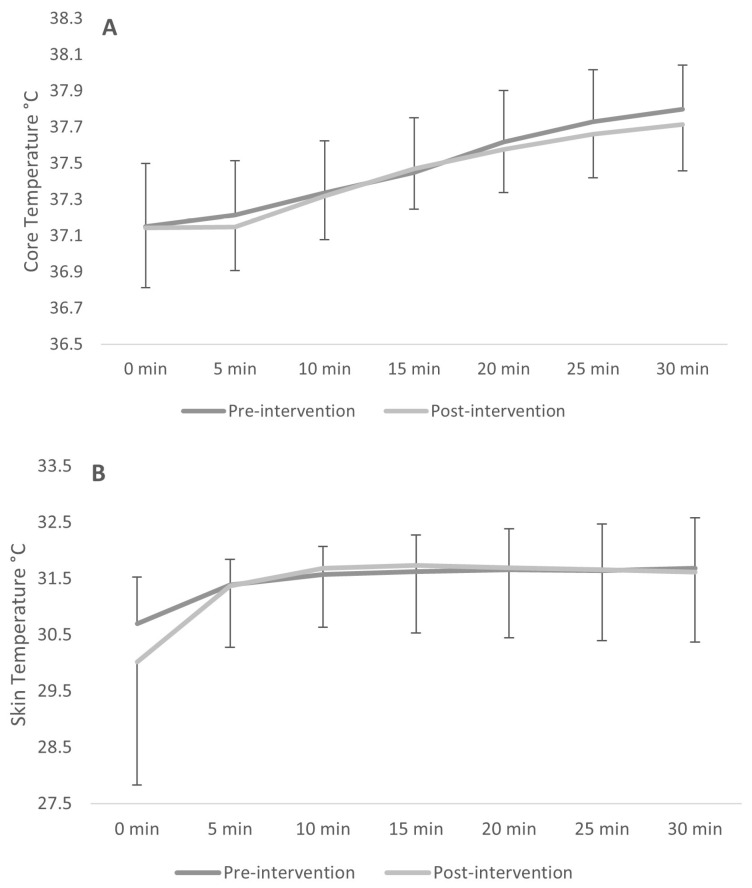
(**A**): Mean ± SD gastrointestinal (core) temperature over time pre-intervention and post-intervention. (**B**): mean ± SD skin temperature over time pre-intervention and post-intervention.

A 2 × 7 repeated measures ANOVA revealed a significant main effect of time on gastrointestinal temperature, F(2.29, 18.28) = 62.11, *p* < 0.001, ηp^2^ = 0.886, indicating a general increase in temperature during the exercise bout. Gastrointestinal temperature was lower post intervention (Figure 5), but this effect was not significant, F(1, 8) = 0.26, *p* = 0.622, ηp^2^ = 0.032, and there was no interaction, F(1.64, 13.14) = 0.39, *p* = 0.648, ηp^2^ = 0.046. Similarly, on skin temperature there was a significant main effect of time, F(2.07, 16.56) = 18.75, *p* < 0.001, ηp^2^ = 0.701, but no main effect of intervention, F(1, 8) = 0.08, *p* = 0.786, ηp^2^ = 0.010, and no interaction, F(1.14, 9.12) = 2.11, *p* = 0.180, ηp^2^ = 0.209.

## 4. Discussion

This study investigated the effect of four consecutive days of HWI on physiological and perceptual markers in older adults during moderate intensity cycling. The HA strategy was sufficient to induce increases in core temperature that would elicit physiological adaptation in the majority of participants. The key findings indicate that following STHA participants had an increased exercise capacity whilst exhibiting lower HR values, indicating reduced cardiovascular strain.

The finding that participants were able to produce a higher power output during the 30 min exercise bout following four days of passive HA, with a concomitant reduction in HR, indicates a reduced cardiovascular cost associated with physical activity. The main physiological mechanism underlying these changes is likely an increase in plasma volume (PV). Acute HWI can elicit immediate increases in PV due to fluid shifts from interstitial spaces [29], with repeated exposure over subsequent days resulting in PV expansion [30]. Although both passive and active HA protocols bring about physiological adaptations, passive HA tends to drive central cardiovascular adaptations, rather than peripheral changes (e.g., sweat gland output) as seen following active HA. The findings from the present study are supported, in part, by Rivas et al. [31], who reported that a 10-day active HA protocol resulted in a reduced metabolic energy expenditure when exercise was matched for external work rate. The authors stated that this beneficial change was not wholly due to improved heat dissipation mechanisms, rather reductions in metabolic heat production. The STHA strategy utilised in the present study was considerably shorter (4 days rather than 10); however, this was still sufficient to bring about small but significant changes in HR and power output. HWI has previously been shown to increase HRV in older adults [32] and Grässler et al., [33] highlighted that improvements in exercise capacity and reduced exercise heart rate are indicators of increased cardiovascular stability as determined by heart rate variability in older populations.

As anticipated, both T_skin_ and T_gast_ increased over time during the exercise bout pre- and post-STHA. Participants were permitted to drink ad libitum during exercise, although only 2 of the 9 participants chose to do so (≤150 mL fluid). Both participants ingested the temperature pills at least 5 h before completing their exercise bout (both participants reported taking their pill on waking), and there was no evidence that the acute fluid intake impacted upon T_gast_ measurements. Although there were no statistically significant differences in temperature pre- to post-intervention, it is possible that the study was underpowered to detect small effects. That said, even very small changes in thermoregulatory responses may prove to have clinical significance when attempting to mitigate the negative impacts of increased thermal load associated with extreme ambient temperatures and heatwaves [10]. Although caution should be applied and further work is needed, passive HA could be considered as a non-pharmacological intervention to reduce heat-related hospitalisations during heatwaves due to thermoregulatory adaptations to HA.

Although the findings from this study are promising, several contextual factors should be considered. All participants in the study were current UK residents, but geographic location (and corresponding environmental conditions) would likely impact upon the effectiveness of the intervention. Individuals who are regularly exposed to high temperatures (as a result of climate conditions, or occupational or leisure activities) may already be well adapted, and as a result, less vulnerable to the effects of heatwave conditions. In the present study one participant appeared to be heat acclimated at study onset (evidenced by a much smaller rise in core temperature during the initial immersion, and anecdotal evidence suggesting regular use of hot tubs). As such, previous HA exposure should be considered both in terms of participant vulnerability and anticipated adaptive response.

Regarding the utility of HA strategies for older and vulnerable adults, a number of barriers and considerations have previously been identified. For example, it has been reported that some individuals are unable to maintain the prescribed workloads during active HA strategies [11,13]. As such, this can mean that the core temperature value required to bring about physiological adaptation (38.5 °C) may not be reached, or maintained for a sufficient period of time, during the exercise session. Whilst individuals exhibiting higher aerobic capacity (or VO2 max) values may be better able to undertake higher intensity exercise, those with lower VO2 max values can still benefit from training induced thermoregulatory adaptations [34]. However, where individuals have limited mobility or are otherwise unable to exercise, passive strategies are likely more appropriate. Whilst many studies have utilised purpose-built environmental chambers or saunas [10], these are often not widely accessible and are costly. Therefore, passive HWI interventions offer a low-cost and more readily accessible alternative to traditional methods, particularly for individuals who may not be physically capable of undertaking active isothermic protocols. Notwithstanding, several practical and logistical considerations remain, not least the need for safety monitoring during immersion protocols to protect against and pre-empt adverse events, and potential issues with acceptability and adherence in the target population. Furthermore, passive HWI could be a practical pre-habilitation strategy for elderly patients who may be unable to engage in active HA due to mobility issues. The exercise bout in the present study was completed in thermoneutral, rather than hot conditions, so whilst this does not mimic environmental extremes, the findings still indicate that positive physiological adaptations were evident during exercise-induced hyperthermia. The reduced cardiovascular cost during exercise would likely be apparent during activities of daily living in hot conditions, although further evidence is required to support this hypothesis.

The present study provides important new insight on the benefits of HA, and more specifically passive HWI, in older adults, adding to the scarce literature and providing a starting point for further investigations. It should be reiterated that the participants recruited for this study were healthy and did not have any known contra-indications to the protocol other than being a vulnerable group with regard to age. As such, the findings reported herein cannot be broadly applied to all older individuals who may have additional risk factors that preclude them from undertaking passive HA in this way. Nevertheless, the relatively small sample size should be taken into consideration. The sample size was based on relevant prior STHA studies (i.e., in a total of 12 studies on ageing, participant n = 6–10 [10]), also recognising that the numerous, lengthy visits involved a considerable effort for participants.

Whilst this study offers new knowledge in relation to STHA for vulnerable adults, there are several areas that require further investigation. As already highlighted, the exercise bout utilised in the current study was not undertaken in hot conditions. As such, there is a need to investigate whether the observed adaptations are still evident under different environmental conditions. It is possible that the benefits may be attenuated by additional exogenous heat stress, or conversely, that the beneficial effect of HWI may be more pronounced in hot conditions. Secondly, it is important to understand dose response in relation to HWI, particularly when considering the constraints relating to accurate weather prediction. Whilst four days offered some adaptive benefits, it may be that additional sessions may offer further protection. Thirdly, it is still not entirely clear whether the number of days, or number of exposures, is more important to attain physiological adaptation. By investigating this further, it may be possible to increase the protective effect without increasing the time frame (i.e., 4 days of 2 exposures per day) or by condensing the protocol into fewer days (e.g., 2 days of 2 exposures). Lastly, and possibly most importantly, it is vital to understand the tolerability of HA protocols in this population, and to consider additional risks posed by specific health conditions and medications in order to avoid causing, rather than mitigating harm. It may be that for certain individuals HA strategies, even if passive in nature, may pose greater risks than relying on acute heat mitigation strategies such as cold beverage ingestion, or the use of external cold application (e.g., cold-water immersion, cooling vests, ice packs and wet towels, etc.). By investigating these areas using well controlled interventions, it may be possible to provide best practice guidelines to individuals, practitioners and policy makers, to protect vulnerable individuals from the negative impacts of heat related illness.

## 5. Conclusions

Overall, the findings from this study indicate that four days of passive HWI at 40 °C is sufficient to elicit physiological adaptation in older adults. Specifically, participants exhibited a significant reduction in average heart rate and increase in power output during 30 min of cycling in thermoneutral conditions at an intensity of 75–80% age predicted HR_max_. These findings have implications for the use of STHA by older populations, indicating that it may be an effective strategy to reduce cardiovascular strain, which may reflect a reduced thermal strain during exercise or activities of daily living in vulnerable individuals. Furthermore, passive HA may provide a non-pharmacological intervention to reduce heat-related hospitalisations during heatwaves, and act as a practical pre-habilitation strategy for patients with mobility issues that are unable to engage in more active HA methods.

## Figures and Tables

**Table 1:** Participant characteristics pre-intervention.

N = 9	Mean (SD)
Stature (cm)	165.7 (10.8)
Body mass (kg)	72.2 (19.4)
BMI	26.0 (4.8)
Body fat (%)	28.6 (5.8)

**Table 2:** Measures taken pre and post-intervention.

	Pre-Intervention Mean (SD)	Post-Intervention Mean (SD)
Body mass (kg)		
Pre-exercise	72.2 (19.4)	72.6 (19.3)
Post-exercise	72.1 (19.2)	72.5 (19.3)
Heart rate (bpm)	112 (3)	109 (4) *
Heart rate (% age predicted HR_max_)	78 (1)	76 (2)
Power output (W)	51.33 (14.76)	58.78 (18.23) *
Distance (km)	10.99 (1.24)	11.44 (1.65)
Thermal sensation rating (AU)		
Pre-exercise	3.56 (1.01)	3.44 (0.73)
15 min	5.67 (0.71)	5.66 (0.71)
30 min	6.33 (0.50)	6.00 (0.87)
Thermal comfort rating (AU)		
Pre-exercise	5.22 (1.48)	4.56 (0.88)
15 min	4.78 (1.09)	4.67 (1.12)
30 min	4.56 (1.74)	4.00 (1.58)
Rate of perceived exertion		
15 min	12.11 (1.54)	12.56 (1.67)
30 min	12.56 (1.67)	13.22 (2.11)

* Significantly different between trials.

## Data Availability

The de-identified raw data for all participants is openly available at https://osf.io/5acjt/, accessed on 30 January 2025.

## Supplementary Materials

The following supporting information can be downloaded at: https://www.mdpi.com/article/10.3390/healthcare14081005/s1, Health Screen Questionnaire.

## Abbreviations

The following abbreviations are used in this manuscript:

HA	Heat Acclimation
HWI	Hot Water Immersion
STHA	Short Term Heat Acclimation
TS	Thermal Sensation
TC	Thermal Comfort
T_skin_	Mean Skin Temperature
T_gast_	Gastrointestinal Temperature
RPE	Rating of Perceived Exertion
HR	Heart Rate
PO	Power Output
BMI	Body Mass Index
BF%	Body Fat Percentage
HR_max_	Heart Rate Maximum
PV	Plasma Volume
