# Supplementary material for: Four-Days of Passive Heat Acclimation Increases Exercise Capacity in Healthy Older Adults Living in the UK
Source: Healthcare (Basel). 2026 Apr 11;14(8):1005. doi: 10.3390/healthcare14081005 (PMC13116818; doi:10.3390/healthcare14081005)
Supplement: Supplementary file 1 [file healthcare-14-01005-s001.zip › healthcare-4177365-supplementary.pdf]

Middlesex University
West Stand
StoneX Stadium,
Greenlands Lane,
London,
NW4 1RL

Middlesex University
London Sport Institute

Health Screen Questionnaire

Please answer the following questions. If you have any doubts or difficulty with the questions, please ask the investigator for guidance. These questions are to determine whether the proposed exercise is appropriate for you. Your answers will be kept strictly confidential.

1. Are you: Male or Female (please circle)
2. What is your date of birth?

Day: __________ Month: __________ Year: __________ and Age: ______ years

3. When did you last visit your doctor (please circle)? In the:

Last week   Last Month   Last Six Months

Last Year   Over a year ago

	YES	NO
**4.** Are you accustomed to regular moderate intensity exercise?		
**5.** Are you currently taking any medication?		
**6.** Has your doctor ever advised you not to take vigorous exercise?		
**7.** Has your doctor ever said “you have heart trouble”?		
**8.** Has your doctor ever said “you have high blood pressure”?		
**9.** Have you ever taken medication for blood pressure or your heart?		
**10.** Do you feel pain in your chest when you undertake physical activity?		
**11.** In the last month have you had pains in your chest when not doing any physical activity?		
**12.** Do you have a cardiac pacemaker or other implanted electromedical device?		
**13.** Has your doctor (or anyone else) said “you have raised blood cholesterol”?		
**14.** Have you had a cold or feverish illness in the last month?		
**15.** Do you ever loose balance because of dizziness, or do you ever lose consciousness?		
**16.** Do you suffer from back pain that may be made worse by physical		

activity?		
**17.** Do you suffer from asthma?		
**18.** Do you have any joint or bone problems which may be made worse by physical activity?		
**19.** Has you doctor ever said "you have diabetes"?		
**20.** Have you ever had viral hepatitis?		
**21.** Do you have epilepsy?		
**22.** Do you suffer from any neurological disorders or injuries?		
**23.** Do you suffer from any muscular or ligament disorder or injury?		
**24.** Do you have any intolerances to heat or high temperatures?		
**25.** Do you have any swallowing, gastrointestinal disorders/issues?		
**26.** Have you ever had gastrointestinal surgery?		
**27.** Are you due to undertake an Nuclear Magnetic Resonance (NMR) or Magnetic Resonance Imaging (MRI) scan during the period of the study and within 7 days after the study?		
**28.** Do you know of any reason, not mentioned above, why you should not exercise?		
**Please fill in any other details relevant to the above here:**		

I have completed the questionnaire to the best of my knowledge and any questions I had have been answered to my full satisfaction.

Name (CAPS): ______________________________

Signed: ______________________________

Date: ______________________________

Please Note: If you have answered 'YES' for any questions 4 to 28, you will need to consult your GP prior to any further participation in the study.
